# Leucine-rich repeat neuronal 1 as a prognostic indicator and functional modulator in breast cancer

**DOI:** 10.3389/fonc.2025.1724785

**Published:** 2025-12-11

**Authors:** Jiaxin Wang, Xiqian Zhou, Ding Ping, Meiling Lu, Yue Zhang, Hongming Song, Junyong Zhao, Dengfeng Li

**Affiliations:** 1 Department of Breast and Thyroid Surgery, Shanghai Tenth People’s Hospital, Institute of Breast Disease, Nanjing Medical University, Shanghai, China; 2 School of Medicine, Tongji University, Shanghai, China; 3 Department of Central Laboratory, Shanghai Tenth People’s Hospital of Tongji University, School of Medicine, Tongji University, Shanghai, China; 4 Breast Disease Center, The Affiliated Hospital of Qingdao University, Qingdao, Shandong, China

**Keywords:** leucine-rich repeat neuronal 1, breast cancer, metastasis, Wnt signals, immunotherapy

## Abstract

**Introduction:**

Advanced breast cancer remains a major therapeutic challenge with high mortality rates. While leucine-rich repeat (LRR) proteins play roles in various cancers including breast cancer, their specific functions are still underexplored. This study aimed to address this research gap by focusing on the leucine-rich repeat neuronal (LRRN) family, with the goal of clarifying their expression, clinical value, and prognostic significance in breast cancer—particularly LRRN1 in advanced cases.

**Methods:**

To investigate the expression of LRRN family members in breast cancer, this study utilized public databases including TCGA-BRCA, GEO, and UALCAN. It further analyzed the clinical value of these LRRN family members and specifically identified the prognostic significance of LRRN1 in advanced breast cancer cases. Functional assays (assessing proliferative, migratory, and invasive capabilities of cancer cells) were conducted to evaluate LRRN1’s biological effects. Additionally, WGCNA, GO analysis, KEGG analysis, and western blotting were employed to verify the molecular pathways impacted by LRRN1.

**Results:**

LRRN1 was found to be significantly downregulated in advanced breast cancer tissues compared to normal tissues (p < 0.001). Moreover, high LRRN1 expression correlated with better disease-free survival (DFS) in advanced breast cancer patients (HR=0.755, 95% CI: 0.617–0.923, p < 0.01). Functional assays revealed that LRRN1 suppresses cancer cell metastasis (without affecting cancer cell proliferation) (p<0.01). Results from WGCNA, GO, KEGG analyses, and western blot confirmed that LRRN1 impacts the Wnt signaling pathway (p<0.05). Additionally, LRRN1 may activate γδ T cells and resting dendritic cells, regulate the M1/M2 macrophage balance in the tumor microenvironment (p<0.001), and mediate the efficacy of certain tyrosine kinase inhibitors (TKIs) (p<0.001).

**Discussion:**

Overall, LRRN1 emerges as a promising prognostic indicator and functional mediator in advanced breast cancer. It potentially exerts its effects by influencing the Wnt signaling pathway and regulating immunotherapy responses. However, the specific mechanisms underlying LRRN1’s actions, as well as its potential clinical applications, still require further exploration to fully translate these findings into clinical practice for advanced breast cancer treatment.

## Introduction

New statistics show that by 2025, breast cancer is the most common cancer type and accounts for 32% of all new cancer cases in women and will be the second leading cause of cancer-related deaths in 2025 (1). Fortunately, Screening methods like mammography and magnetic resonance imaging have proven to be useful in detecting breast cancer early, which helps reduce breast cancer mortality (2). However, the identification of more precise and therapeutically relevant biomarkers remains essential for improving early detection and therapeutic responsiveness (3).

In this context, leucine-rich repeat (LRR) proteins—historically associated with neurological function have gained increasing attention. Notably, members of the leucine-rich repeat neuronal (LRRN) protein subfamily—LRRN1, LRRN2, LRRN3, LRRN4, and LRRN4CL—are characterized by dysregulated expression in neuronal tissues and more importantly, they have been increasingly associated with the development of various malignancies (4–8). Predominantly localized at synaptic sites, LRR proteins play crucial roles in modulating synaptogenesis throughout the maturation of the central nervous system as well as in cancer progression (9, 10). Notably, Nod-like receptor protein 3 (NLRP3), a member of the LRR protein family, has been reported to contribute to tumor metastasis, therapeutic resistance, and the regulation of cancer treatment responses (11). In breast cancer, Leucine-rich repeat-containing (LRRC) protein 15 (LRRC15) has been identified as one of the significantly overexpressed genes in tumors that metastasize to the bone (12). Building on our earlier findings that LRRC56 facilitates breast cancer progression, we further explored the potential oncogenic roles of other members from LRR protein family in this malignancy (13).

Among members of LRRN protein families, accumulating evidence has demonstrated that LRRN1 possesses both diagnostic and prognostic potential across various cancer types (14–16). Notably, genetic ablation of LRRN1 suppresses the malignant progression of pancreatic ductal adenocarcinoma through interference with HIF-1α/Notch signaling pathway activation (17). Meanwhile, elevated LRRN1 expression could inhibit apoptosis in gastric cancer by downregulating the Fas/FasL signaling cascade (18). In addition, LRRN1 has been reported to correlate with other genes, including SNCG, GAMT, and PDE1B, collectively exhibiting prognostic value in gastric cancer (9). However, a critical gap remains: Although studies investigating LRRN1 in breast cancer remain limited, exploring the functions of the LRRN protein family may facilitate the identification of novel therapeutic targets.

To address these knowledge gaps, our study used data from The Cancer Genome Atlas (TCGA-BRCA) and the Gene Expression Omnibus (GEO) to comprehensively analyze the expression profiles of the LRRN gene family, and to evaluate their correlations with clinical characteristics and patient prognosis in breast cancer. During our analysis, we found that LRRN1 expression demonstrated a significant association with key pathological characteristics of breast cancer and exhibited potential clinical relevance throughout the analysis. We expect that our findings could facilitate the development of novel therapeutic strategies for breast cancer treatment.

## Materials and methods

### Datasets collection

To find new targeted treatments for metastatic breast cancer, we conducted a search in the ONCOMINE database using the keyword “breast cancer metastasis”. Clinical and prognostic information for all participants in this study was obtained from TCGA (https://portal.gdc.cancer.gov) and GEO (https://www.ncbi.nlm.nih.gov/gds/) publicly accessible databases. Transcriptome sequencing profiles and corresponding clinical data were obtained from 1,231 breast cancer patients included in the TCGA-BRCA cohort. To check our findings, we used two separate microarray datasets, GSE21653 and GSE65194, from the GEO database. All data utilized in this study are de-identified and publicly available, thereby conforming to ethical guidelines and data-sharing regulations.

### Pan-cancer expression analysis and clinical features of LRRNs in breast cancer

Transcriptomic profiles of LRRN family genes were comprehensively analyzed across 33 cancer types via the UALCAN (University of Alabama at Birmingham Cancer Data Analysis Portal) database (19, 20) (https://ualcan.path.uab.edu/cgi-bin/ualcan-res.pl). Expression differences were evaluated by comparing tumor tissues with their matched normal counterparts and across diverse cancer subtypes. In breast cancer specifically, LRRN gene expression was compared between malignant and normal breast tissues. The associations between gene expression profiles and key clinicopathological parameters—such as patient age, tumor size (T), lymph node metastasis (N), distant metastasis (M), and American Joint Committee on Cancer (AJCC) stage—were systematically investigated. Visualization of expression patterns was performed using the R package “ggplot2,” and results were presented as boxplots.

### Prognostic value assessment of LRRN1 expression in breast cancer

Based on the median expression level of LRRN1, patients were divided into high and low expression groups. Survival analysis was conducted using the Kaplan–Meier Plotter (21, 22) (https://kmplot.com) to evaluate the prognostic significance of LRRN family gene expression in relation to overall survival (OS) in breast cancer patients. To assess the independent prognostic significance of LRRN1, a multivariate Cox proportional hazards regression model was constructed using clinical parameters and gene expression profiles obtained from the TCGA-BRCA dataset to assess independent prognostic factors.

### Functional enrichment and co-expression network analysis

To investigate the potential biological functions of LRRN1 in breast cancer, Pearson correlation analysis was conducted to identify genes whose expression patterns were significantly correlated with LRRN1. To explore the biological processes and signaling pathways linked to LRRN1, we selected the top 100 genes showing the strongest correlations for Gene Ontology (GO) and Kyoto Encyclopedia of Genes and Genomes (KEGG) enrichment analyses. Furthermore, Gene Set Enrichment Analysis (GSEA) was performed after dividing the samples into high and low LRRN1 expression groups according to the median expression level. This approach aimed to identify significantly enriched molecular functions and signaling pathways linked to differential LRRN1 expression.

To delve deeper into the co-expression patterns, we employed Weighted Gene Co-expression Network Analysis (WGCNA) using the transcriptomic data from the TCGA-BRCA dataset. Through hierarchical clustering based on pairwise expression similarity, genes were organized into co-expression modules. Modules exhibiting similar expression profiles were then merged. Subsequently, module–trait correlation analyses were performed to evaluate the relationships between each module and key clinical features, including patient age, tumor size, lymph node metastasis, distant metastasis, and AJCC stage.

### Immune landscape characterization and drug sensitivity evaluation

To investigate the association between LRRN1 expression and the tumor immune microenvironment, transcriptomic profiles from the GSE65194 dataset were analyzed. Patients were categorized into high and low LRRN1 expression groups according to the median expression values. Immune scores were meticulously calculated using functions from the estimate package in R, this package initially filters the input gene expression data to isolate the “signature gene sets” essential for the ESTIMATE algorithm—usually encompassing marker genes for stromal and immune cells—and subsequently computes StromalScore, ImmuneScore, and ESTIMATEScore for each individual sample. These calculated scores were then Compared between high and low LRRN1 groups to assess immune infiltration levels. The CIBERSORT algorithm was utilized to quantify the relative abundance of 22 distinct immune cell types within the tumor microenvironment (TME). Immune cell composition was then quantified and visualized to elucidate differences in immune infiltration patterns between separate LRRN1 expression subgroups. Furthermore, differential expression analysis was performed on key immune checkpoint-related genes to explore potential immunoregulatory differences. The results were visualized using the “ggplot2” program.

In addition, to assess potential therapeutic implications, drug sensitivity analysis was carried out using data from the Genomics of Drug Sensitivity in Cancer (GDSC) database (https://www.cancerrxgene.org/). The half-maximal inhibitory concentration (IC50) values of various chemotherapeutic and targeted agents were compared between high and low LRRN1 expression groups to predict differences in drug responsiveness.

### Cell culture and plasmid transfection

MDA-MB-231 and MCF-7 breast cancer cells were cultured in Dulbecco’s Modified Eagle Medium (DMEM; WISENT, Canada) supplemented with 10% fetal bovine serum (FBS; Absin, Shanghai, China) and 1% penicillin-streptomycin (PS; Beyotime, Shanghai, China), and maintained at 37 °C with 5% CO_2_. When cells reached 70–80% confluence in 6-well plate, plasmid transfection was performed using Lipofectamine 8000 (Beyotime, Shanghai, China). The LRRN1 expression plasmid was purchased from Miaoling Plasmid Biotechnology Co., Ltd. (Wuhan, China).

### RNA extraction and real-time quantitative polymerase chain reaction

Total RNA was extracted from MDA-MB-231 and MCF-7 breast cancer cells using an RNA extraction kit (Beyotime, Shanghai, China) following the manufacturer’s protocol (13). The isolated RNA was then reverse-transcribed into complementary DNA (cDNA) using the HiScript III 1st Strand cDNA Synthesis Kit with gDNA Wiper (Vazyme, Nanjing, China) (13). RT-qPCR was performed using gene-specific primers, with GAPDH serving as an internal control. The relative mRNA expression levels were calculated using the 2^−ΔΔCt^ method. The sequences of primers are: LRRN1: Forward 5’-3’: GCGTATGTGAAATTCGTCCCT; Reverse 5’-3’: TCCTTGTTAAGCGGAGGTCAT. GAPDH: Forward 5’-3’: GGAAGCTTGTCATCAATGGAAATC; Reverse 5’-3’: TGATGACCCTTTTGGCTCCC.

### Proliferation assays

After transfection, MDA-MB-231 and MCF-7 cells were trypsinized, resuspended, and seeded into 96-well plates at a density of 2×10^3^ cells/well. Cell viability was assessed every 24 hours for 5 consecutive days using the MTT (3-(4,5-Dimethylthiazol-2-yl)-2,5-diphenyltetrazolium bromide assay (Beyotime, Shanghai, China), with the cell culture medium remaining unchanged throughout the entire 5-day period. Absorbance measured at 490 nm to generate proliferation curves. For colony formation assays, transfected cells were plated in 12-well plates at 800 cells/well and incubated under standard conditions for 7–10 days. Colonies were fixed in 4% paraformaldehyde (PFA; Servicebio, Wuhan, China) and stained with 0.1% crystal violet (Yeasen, Shanghai, China) for visualization.

### Wound healing assay

Transfected MDA-MB-231 cells (250,000 cells per well) were plated in 12-well plates and allowed to grow until they achieved approximately 90% confluence. A linear scratch was introduced into the monolayer using a sterile 200 μL pipette tip to create a uniform wound. Following this, the medium was changed to DMEM supplemented with 3% fetal bovine serum (FBS). The process of wound healing was monitored and imaged at 0, 24, 48, and 72 hours post-scratch. The rate of cell migration was assessed quantitatively by measuring the reduction in wound width over the observation period.

### Transwell migration and invasion assays

MDA-MB-231 cells were prepared as single-cell suspensions (200 μL) in serum-free DMEM and seeded into the upper chambers of 24-well Transwell inserts (Corning, NY, USA). For migration assays, inserts without coating were used, whereas for invasion assays, inserts coated with Matrigel (Yeasen, Shanghai, China) were applied. The lower chambers were filled with 500 μL of DMEM containing 10% FBS to serve as a chemoattractant. After incubation for 16 hours, non-migratory or non-invasive cells on the upper surface were removed with Phosphate Buffered Saline (PBS, Servicebio, Wuhan, China). Cells that penetrated the membrane were fixed with 4% PFA and stained with 0.1% crystal violet. Subsequently, the stained cells on the lower surface were imaged under a microscope, and the number of cells was quantified in representative fields.

### Protein extraction and western blotting

Forty-eight hours following LRRN1 plasmid transfection, protein samples were collected in accordance with our previously described methods (23). Protein samples (40 μg each) were subjected to SDS-PAGE using reagents from Beyotime (Shanghai, China) and subsequently transferred onto 0.45 μm nitrocellulose membranes (Cytiva, MA, USA). The membranes were blocked with 5% non-fat milk for 1 hour at room temperature, followed by overnight incubation at 4 °C with primary antibodies. After three washes with Tris-Buffered Saline-Tween-20 (TBST, Epizyme, Shanghai, China), the membranes were incubated with HRP-conjugated secondary antibodies for 1 hour at room temperature. Protein bands were detected using enhanced chemiluminescence reagent (Yeasen, Shanghai, China). The antibody information were list as below: MMP2(1:1000, Proteintech, 66366-1-Ig, Wuhan, China), MMP9(1:1000, Proteintech, 10375-2-AP, Wuhan, China), FAK(1:1000, Proteintech, 66258-1-Ig, Wuhan, China), WNT5a/b(1:1000, 2530s, Cell signaling Technology, Massachusetts, USA), GAPDH(1:1000, gb15004-100,Servicebio, Wuhan, China), HRP-conjugated Goat anti-Rabbit or anti-Mouse IgG (H+L) (1:4000, AS014, Abclonal, Wuhan, China).

### Statistical analysis

All statistical analyses were performed using R software (version 4.4.2; R Foundation for Statistical Computing, Vienna, Austria), SPSS software (version 20.0; IBM Corp., Armonk, NY, USA), and GraphPad Prism (version 6.0; GraphPad Software Inc., San Diego, CA, USA). Based on the results of the Shapiro-Wilk normality test, we selected either a parametric or a non-parametric test for the comparison among the test groups. The Wilcoxon rank-sum test was applied to assess differences between two groups. The chi-square test was used for comparisons of categorical variables. Univariate and multivariate Cox proportional hazards regression analyses were conducted to identify prognostic factors, with the multivariate model adjusted for relevant clinical covariates. For cell-based experiments, data are presented as the mean ± standard deviation (SD) from at least three independent experiments. Group comparisons were conducted using unpaired Student’s t-test or two-way analysis of variance (ANOVA), as appropriate. A *p*-value < 0.05 was considered statistically significant.

## Results

### Expression of the LRRN family in pan-cancer

To find potential target genes in metastatic breast cancer, we used the ONCOMINE database and found a list of genes linked to breast cancer metastasis (**Supplementary Table 1**). In our earlier work, we discovered that LRR proteins are very important in breast cancer metastasis (**Supplementary Table 2**). Among these proteins, LRRN family members exhibited particularly notable expression patterns in our dataset. To systematically evaluate the potential oncogenic value of the LRRN family across various malignancies, we performed a comprehensive analysis in multiple cancer types using the UALCAN database.

**Figure 1:**
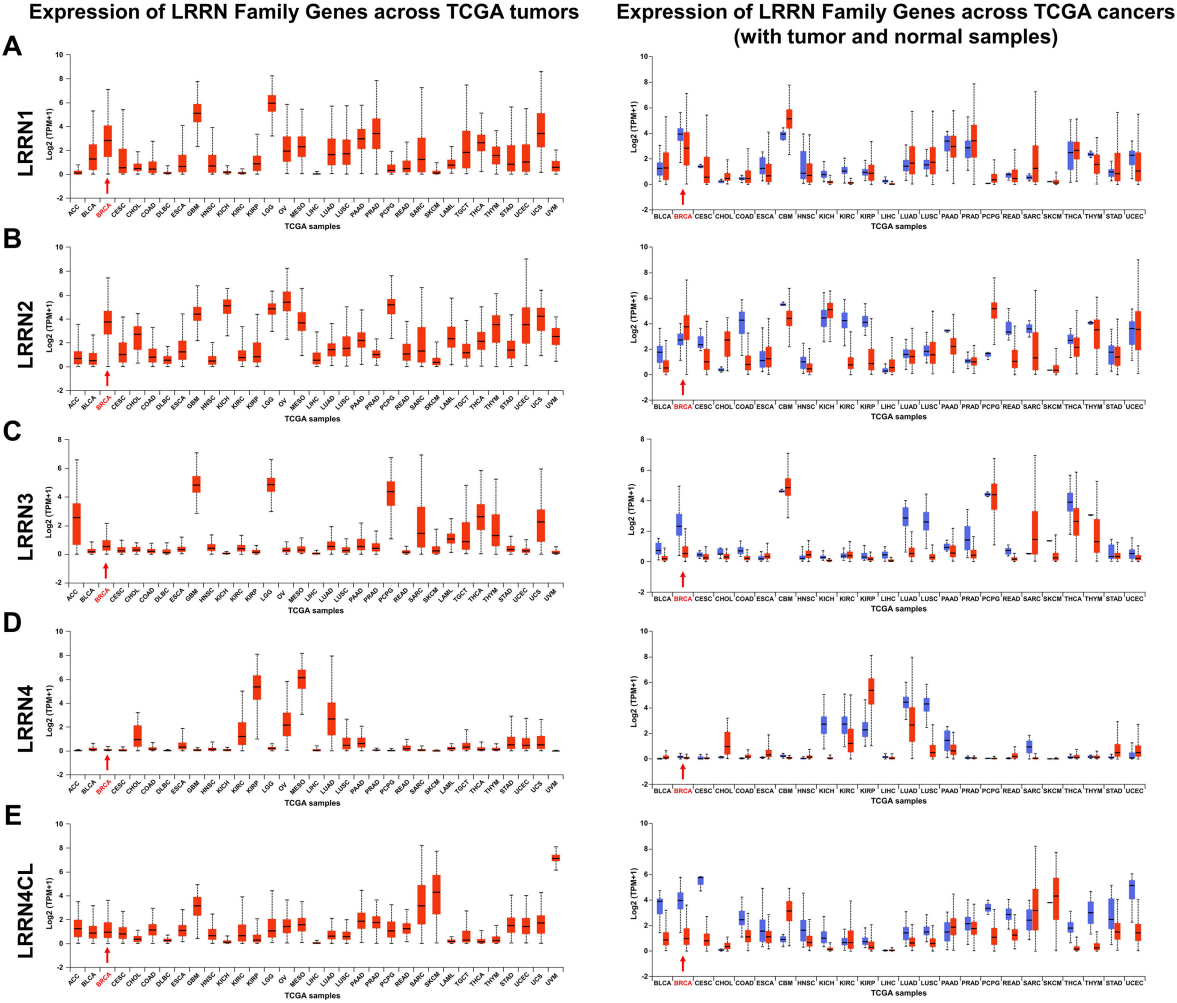
Expression of LRRN family members in various tumors. Pan-cancer analysis of LRRN1 **(A)**, LRRN2 **(B)**, LRRN3 **(C)**, LRRN4 **(D)**, and LRRN4CL **(E)** expression in the TCGA database, comparing tumor and normal samples.

As illustrated in **Figure 1**, distinct expression patterns of LRRN family members were observed across different cancer types. Notably, our analysis revealed significant downregulation of LRRN1, LRRN3, and LRRN4CL in breast cancer tissues (**Figures 1A, C, E**). In contrast, LRRN2 showed marked overexpression in breast cancer specimens (**Figure 1B**). Interestingly, LRRN4 expression did not demonstrate significant differential expression in breast cancer (**Figure 1D**). These findings suggest that different LRRN family members may exert distinct functional roles in breast cancer pathogenesis. The observed differential expression patterns, particularly the consistent downregulation of LRRN1, LRRN3, and LRRN4CL coupled with LRRN2 upregulation, prompted us to focus our subsequent investigations on elucidating the specific mechanisms of these proteins in breast cancer metastasis.

### LRRN family expression in breast cancer and clinicopathological correlations

**Figure 2:**
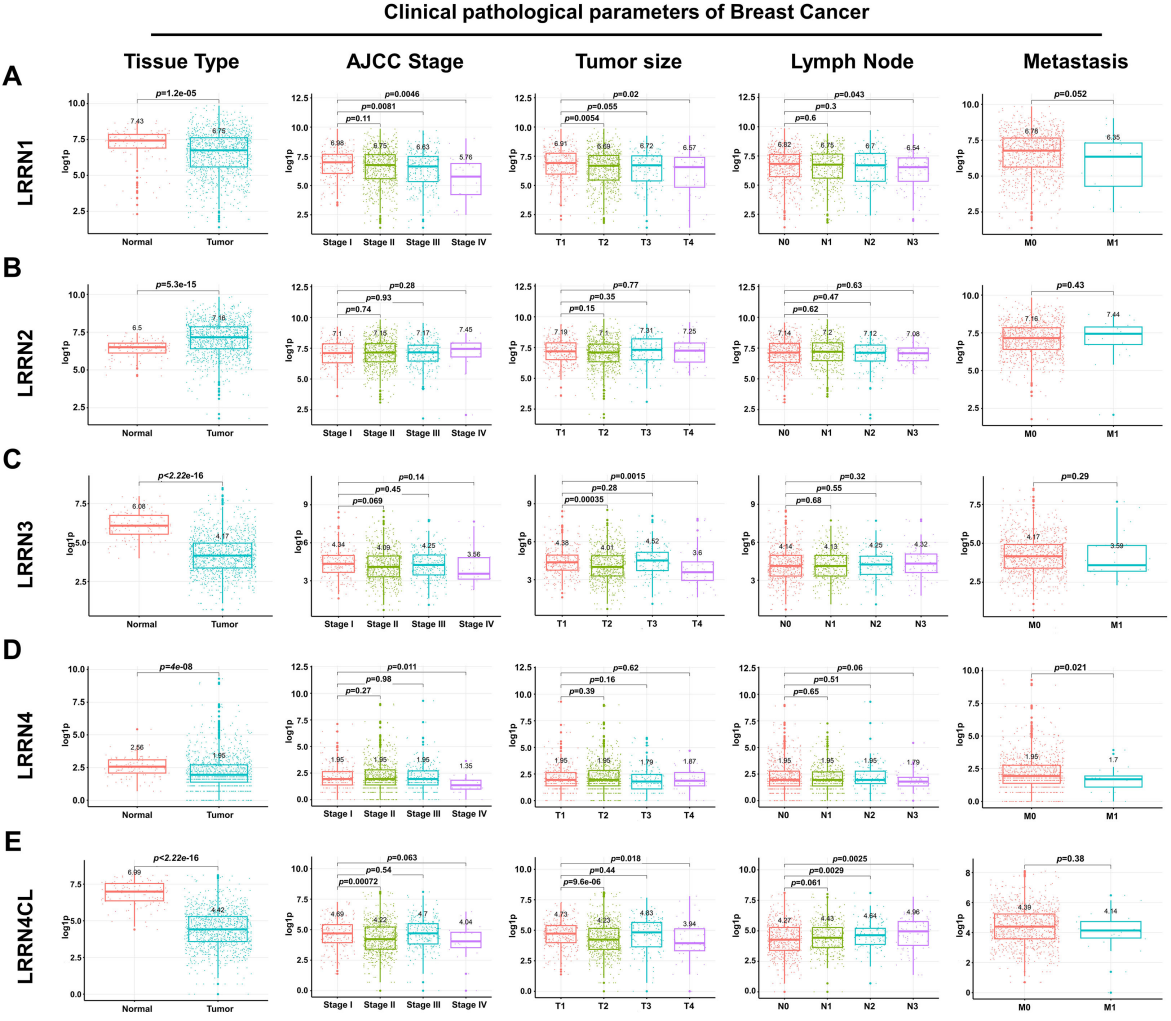
Correlation between LRRN family members and clinical features of breast cancer in the TCGA database. Correlation analysis of LRRN1 **(A)**, LRRN2 **(B)**, LRRN3 **(C)**, LRRN4 **(D)**, and LRRN4CL **(E)** with clinical features including tissue type, AJCC stage, tumor size, lymph node metastasis, and metastasis.

To comprehensively evaluate the clinical significance of LRRN family members in breast cancer pathogenesis, we performed a systematic analysis of their expression patterns and associations with key clinicopathological parameters, including AJCC staging, tumor dimensions, lymph node metastasis, and metastatic dissemination. Our analysis revealed LRRN1’s expression was lowly expressed in cancer tissues (**Figure 2A**). Quantitative assessment demonstrated statistically significant inverse correlations between LRRN1 expression levels and advanced AJCC stage, larger tumor size, and lymph node metastasis. And the association between LRRN1 expression and distant organ metastasis was observed to be following a trend of inverse proportionality with lower LRRN1 expression in metastatic cases. In contrast to LRRN1’s expression profile, LRRN2 exhibited significant upregulation in cancer tissues, though its associations with AJCC stage and tumor size showed only marginal trends without statistical significance. Among other family members, LRRN3, LRRN4, and LRRN4CL were uniformly downregulated in breast cancer tissues. Notably, LRRN4 expression showed significantly lower levels in metastatic cases compared to non-metastatic patients (**Figure 2D**, p = 0.021), mirroring its inverse correlation with stage IV disease. LRRN4CL expression patterns demonstrated significant associations with both tumor size and lymph node metastatic burden (**Figure 2E**), while also exhibiting a progressive decline with advancing disease stage.

These results collectively underscore the distinct clinical significance of different LRRN family members in the progression of breast cancer. The consistent downregulation of LRRN1 across multiple pathological parameters, coupled with its strong inverse correlations with disease progression markers, positions it as a particularly promising candidate for further mechanistic investigation and potential therapeutic targeting in advanced breast cancer.

### Prognostic value of LRRN family in breast cancer

**Figure 3:**
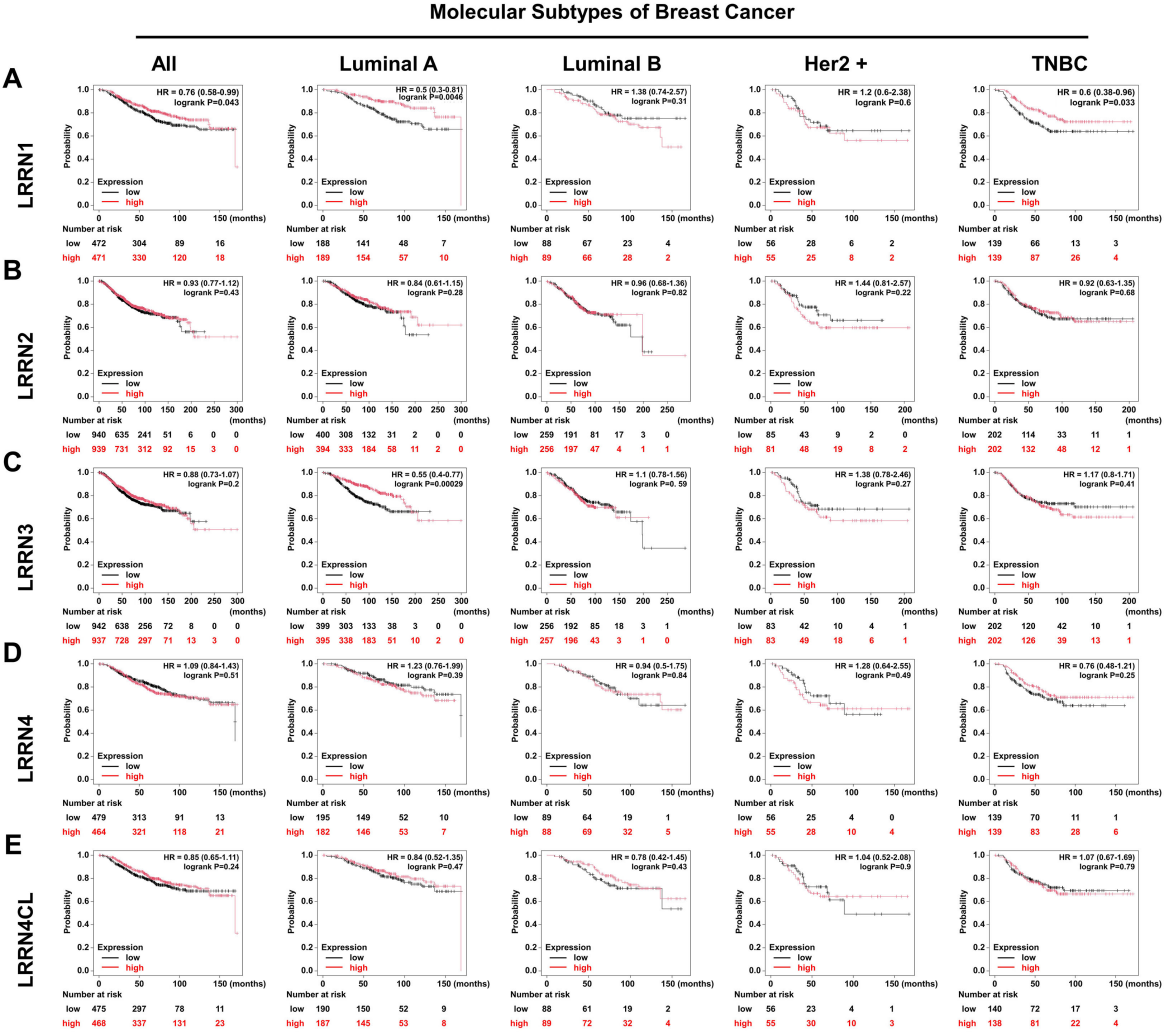
Survival analysis of LRRN family members in different subtypes of breast cancer. Kaplan-Meier survival analysis of LRRN1 **(A)**, LRRN2 **(B)**, LRRN3 **(C)**, LRRN4 **(D)**, and LRRN4CL **(E)** across various breast cancer subtypes, including all types, Luminal A, Luminal B, HER2+, and TNBC, according to the KM-Plotter database.

To elucidate the prognostic implications of the LRRN gene family in breast cancer, we conducted a comprehensive survival analysis using the KM-plotter database (22) to evaluate associations between LRRN expression profiles and clinical outcomes. As illustrated in **Figure 3A**, elevated LRRN1 expression demonstrated a significant correlation with adverse prognosis in breast cancer patients. Subsequent stratification by molecular subtype revealed that elevated LRRN1 expression was associated with favorable survival outcomes in Luminal A and triple-negative breast cancer (TNBC) subtypes, whereas no statistically significant associations were observed in Luminal B or HER2-enriched subtypes (**Figure 3A**). Notably, evaluation of other LRRN family members (LRRN2, LRRN3, and LRRN4) did not demonstrate significant prognostic relevance in the KM-Plotter cohort (**Figures 3B-E**).

In an effort to identify clinically relevant factors within breast cancer, we systematically interrogated multiple breast cancer databases, highlighting LRRN1 as a particularly significant biomarker. We then conducted an in-depth investigation of LRRN1’s prognostic utility. Multivariate analysis revealed statistically significant correlations between LRRN1 expression levels and key clinicopathological parameters, including tumor size (*p* = 0.003), lymph node metastasis (*p* = 0.049), distant metastasis (*p* = 0.034), and advanced pathological stage (*p* = 0.002) (**Table 1**). Our analyses were further confirmed by an independent GEO data (GSE21653), where LRRN1 expression patterns exhibited significant associations with both survival status (*p* = 0.009) and molecular subtypes (*p* = 0.014) (**Table 2**). Univariate Cox proportional hazards regression analysis identified LRRN1 as a protective factor for disease-free survival (DFS) (HR = 0.7486, 95% CI = 0.617-0.923, *p* = 0.006). Importantly, multivariate Cox regression analysis, incorporating established prognostic variables (age, ER/PR/HER2 status, molecular subtype, tumor grade, Ki67 index, and p53 status), confirmed LRRN1 as an independent predictor of DFS (HR = 0.7560, 95% CI: 0.559-0.866, *p* = 0.001) (**Table 3**). These findings highlight the potential clinical utility of LRRN1 prognosis in breast cancer management.

**Table 1:** The correlation of LRRN1 expression and clinical features in TCGA databases.

Clinical pathological parameters		Expression of LRRN1(75%)		χ^2^	*p*
		High (n=683)	Low (n=228)		
Age	≤60	405	116	4.95	**0.026**
	>60	278	112		
Censor	Alive	589	194	0.187	0.665
	Dead	94	34		
Tumor size	T1	196	40	13.769	**0.003**
	T2	394	145		
	T3	73	30		
	T4	20	13		
Lymph Node metastasis	N0	351	100	3.879	**0.049**
	NX	332	128		
Metastasis	M0	674	220	4.481	**0.034**
	M1	9	8		
AJCC Stage	I	134	26	14.907	**0.002**
	II	400	131		
	III	140	63		
	IV	9	8		

A *p* value below 0.05 was considered significant and highlighted (bold).

**Table 2:** The correlation of LRRN1 expression and clinical pathological parameters in breast cancer patients.

Clinical pathological parameters		Expression of LRRN1		χ^2^	*p*
		High (n=73)	Low (n=74)		
Age	≤60	47	35	4.349	**0.037**
	>60	26	39		
Censor	Alive	57	43	6.741	**0.009**
	Dead	16	31		
ER	–	31	30	0.056	0.813
	+	42	44		
PR	–	33	36	0.175	0.676
	+	40	38		
Her2	–	67	66	0.286	0.593
	+	6	8		
Ki-67	low expression	26	16	3.527	0.06
	High expression	47	58		
Molecular Subtype	Luminal A	33	24	10.548	**0.014**
	Luminal B	9	21		
	Her2-Enriched	3	9		
	Basal-like	28	20		
Histological grades	i	11	8	2.38	0.304
	ii	21	30		
	iii	41	36		
p53	–	44	48	0.331	0.565
	+	29	26		

A *p* value below 0.05 was considered significant and highlighted (bold).

**Table 3:** Univariate and multivariate Cox proportional hazards regression analyses for DFS of LRRN1 and pathological features.

Variables	Univariable Cox			Multivariable Cox		
	HR	95.0% CI	*p* value	HR	95.0% CI	*p* value
LRRN1	0.755	0.617-0.923	**0.006**	0.696	0.559-0.866	**0.001**
Age	0.993	0.972-1.015	0.537	0.988	0.966-1.010	0.286
ER	0.756	0.423-1.350	0.344	0.223	0.062-0.800	**0.021**
PR	1.031	0.578-1.840	0.917	2.941	0.877-9.867	0.081
Her2	1.054	0.377-2.946	0.92	1.020	0.351-2.962	0.971
Subtype	1.071	0.858-1.338	0.544	0.796	0.513-1.235	0.308
Grade	1.291	0.852-1.957	0.228	1.429	0.797-2.564	0.231
Ki-67	1.300	0.685-2.467	0.423	0.745	0.313-1.776	0.507
p53	1.329	0.740-2.386	0.342	1.261	0.654-2.431	0.488

A *p* value below 0.05 was considered significant and highlighted (bold).

HR, hazard ratio; CI, confidence interval.

#### LRRN1 suppressed breast cancer cellular metastasis

**Figure 4:**
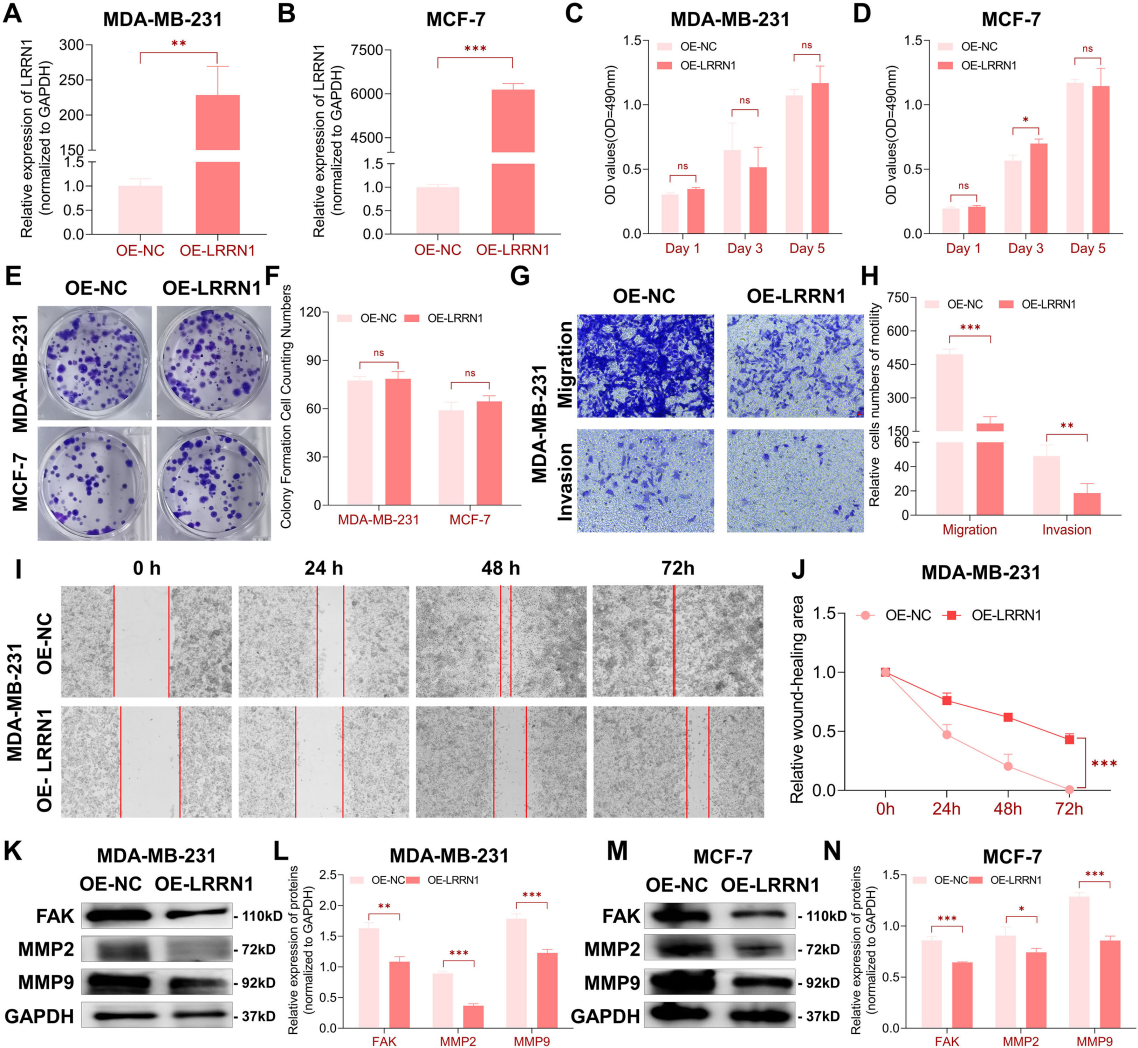
Effects of LRRN1 overexpression on breast cancer cellular migration and invasion. **(A, B)** The transfection efficiency of plasmids of LRRN1 in breast cancer cells confirmed by RT-qPCR. **(C, D)** Overexpression of LRRN1 did not significantly impact cell proliferation in MDA-MB-231 and MCF-7 cells. **(E, F)** Overexpression of LRRN1 had no effect on colony formation in MDA-MB-231 and MCF-7 cells. **(G, H)** Overexpression of LRRN1 reduced migration and invasion of MDA-MB-231 cells, as shown by transwell assays. **(I, J)** Overexpression of LRRN1 decreased migration of MDA-MB-231 cells, as demonstrated by scratch wound assays. **(K-N)** Western blot analysis revealed reduced levels of FAK, MMP2, and MMP9 in LRRN1-overexpressing cells compared to controls, with GAPDH serving as a loading control. ns, no significance, *p < 0.05, **p < 0.01, ***p < 0.001.

Building upon our previous findings that identified LRRN1 as a clinically significant factor in breast cancer, we proceeded to investigate its functional role in malignant progression. We overexpressed LRRN1 in breast cancer cells (**Figures 4A, B**) and evaluated its biological impact. Surprisingly, the proliferation assays demonstrated that LRRN1 overexpression had no significant impact on breast cancer cell proliferation (**Figures 4C-F**). However, transwell migration and invasion assays showed that LRRN1 overexpression significantly suppressed the migratory and invasive capacities of breast cancer cells (**Figures 4G, H**). These findings were further corroborated by wound healing assays, where LRRN1-expressing cells exhibited markedly reduced motility compared to controls (**Figures 4I, J**).

To uncover the molecular mechanisms of these phenotypic changes, we conducted western blot experiments targeting key metastasis-associated proteins. LRRN1 overexpression consistently downregulated the protein expression of focal adhesion kinase (FAK), matrix metalloproteinase-2 (MMP2), and matrix metalloproteinase-9 (MMP9) in both breast cancer cellines (**Figures 4K-N**). These proteins have been previously established as critical mediators of breast cancer metastasis (24). Collectively, our findings suggest that LRRN1 inhibits breast cancer metastasis by regulating the expression of key metastasis-associated proteins.

#### LRRN1 regulated Wnt signal pathway in breast cancer

**Figure 5:**
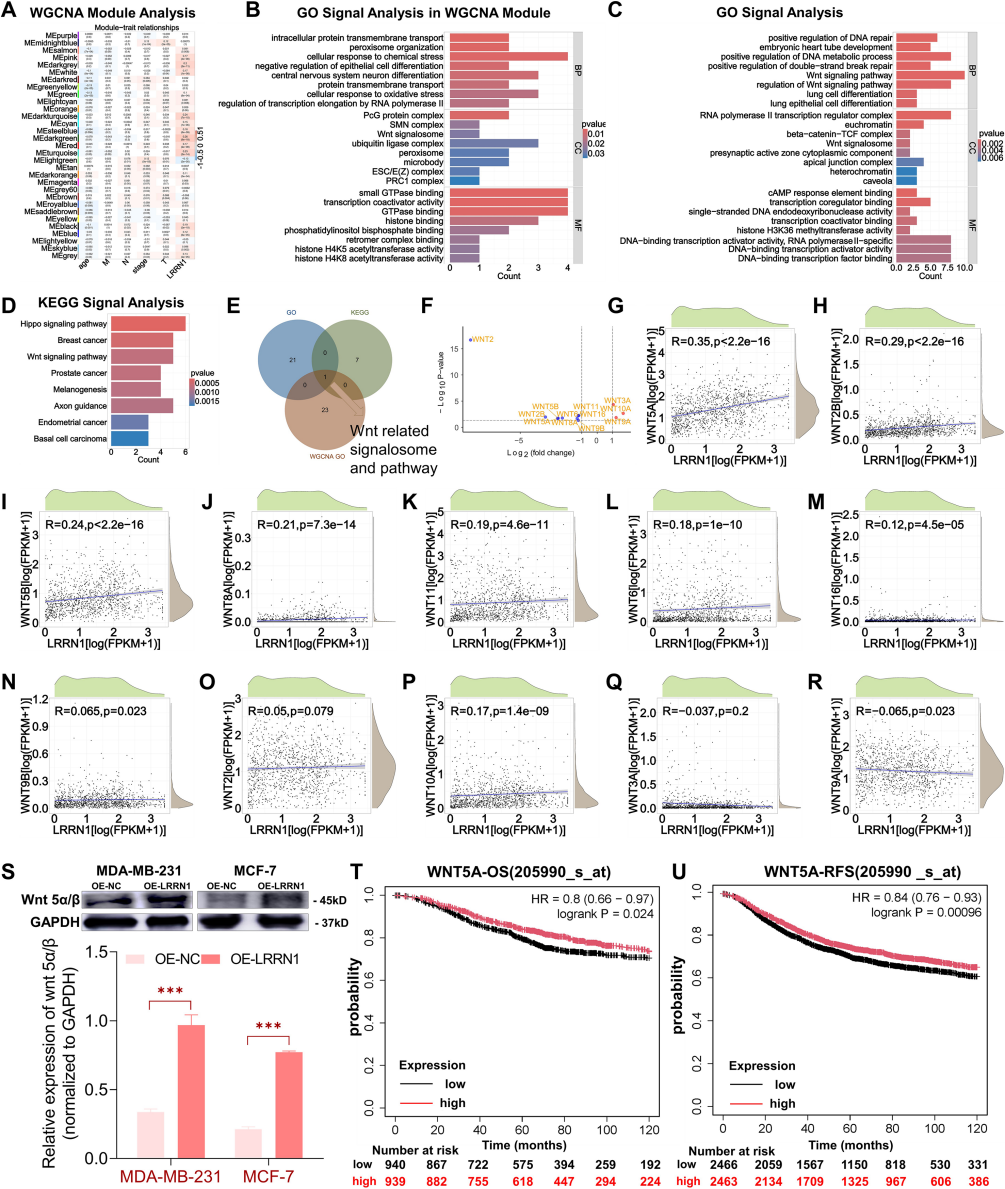
Identification of related genes, pathways, and cellular functions of LRRN1. **(A)** Association between gene expression modules and clinicopathologic characteristics with LRRN1 expression in the TCGA database. **(B)** GO analysis of the MEdarkgreen module. **(C, D)** GO and KEGG analyses of LRRN1 in the TCGA database. **(E)** Intersection of enriched pathways identified through WGCNA, GO and KEGG highlighting key pathways associated with LRRN1. **(F)** Volcano plot depicting differentially expressed Wnt family genes in breast cancer, as identified in the TCGA database. **(G-R)** Correlations between LRRN1 and Wnt family members. **(S)** Western blot analysis of Wnt5 α/β protein levels in LRRN1-overexpressing cells compared to control cells, with GAPDH as a loading control. **(T, U)** Survival analysis of Wnt5α in BC, including OS and RFS. ***p < 0.001.

To elucidate the molecular mechanisms of LRRN1, we conducted WGCNA using data from the TCGA-BRCA cohort. The MElightgreen module exhibited the most robust positive correlation with LRRN1 expression (**Figure 5A**). GO enrichment analysis of this module highlighted pathways related to cellular response to chemical stress, intracellular protein transmembrane transport, ubiquitin ligase complex, Wnt signalosome, and others (**Figure 5B**). Additionally, Pearson correlation analysis (|R| > 0.5, p < 0.05) was performed in the TCGA database to identify genes most strongly correlated with LRRN1. The top 100 correlated genes were then subjected to GO and KEGG pathway analyses (**Figures 5C, D**). The biological processes most closely associated with LRRN1 included the Wnt signaling pathway, positive regulation of DNA metabolic processes, and others. KEGG analysis revealed major pathways such as the Wnt and Hippo signaling pathway. The intersection of these enriched pathways identified key Wnt-related signalosomes and pathways (**Figure 5E**).

We subsequently interrogated the breast cancer metastasis database (**Supplementary Table 1**) to identify potential Wnt-related genes. We found that a series of Wnt-related genes were differentially expressed in the breast cancer metastasis group (**Figure 5F**). We assessed then the correlation between these genes and LRRN1 in breast cancer (**Figures 5G-R**). The results indicated that Wnt5A exhibited a significant positive correlation with LRRN1 in breast cancer (**Figure 5G**). Western blot validation confirmed that overexpression of LRRN1 increased Wnt5A protein levels in two breast cancer cells (**Figure 5S**). Furthermore, survival analysis demonstrated that Wnt5A was correlated with improved OS and recurrence-free survival (RFS). Collectively, these findings support our hypothesis that LRRN1 inhibits breast cancer metastasis through modulation of the Wnt signaling pathway.

#### LRRN1 exhibited the significant value for breast cancer treatment

**Figure 6:**
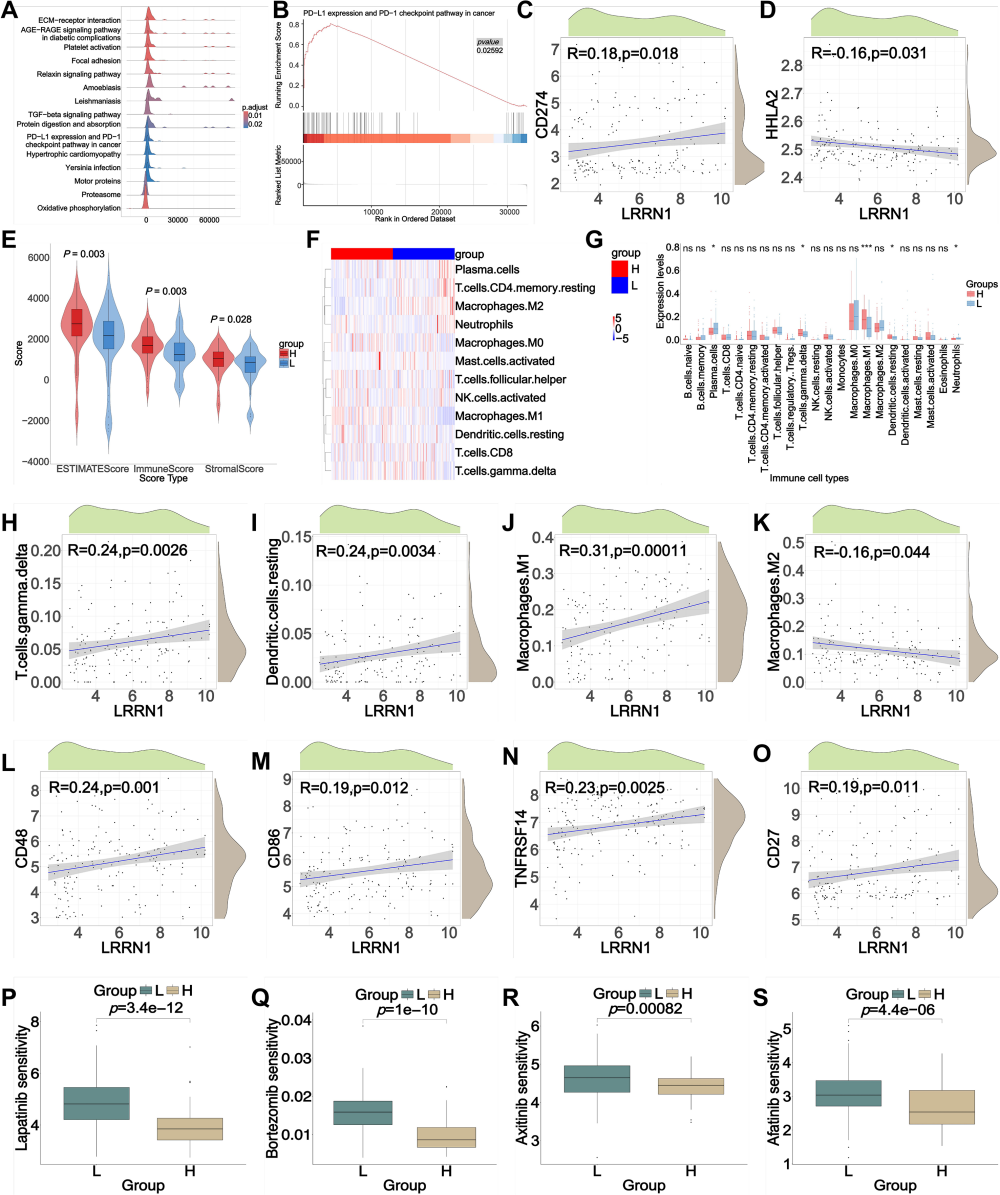
Association of LRRN1 expression with tumor immunity in breast cancer. **(A, B)** Gene Set Enrichment Analysis (GSEA) results and detailed pathways for LRRN1 expression in the TCGA database. **(C, D)** Correlation between LRRN1 expression and immune checkpoints CD274 **(C)** and HHLA2 **(D)** in the GSE65194 dataset. **(E)** Relationships between LRRN1 expression and immune score, stromal score, and estimate score in the GSE65194 dataset. **(F, G)** Comparison of immune cell infiltration abundance and specific immune cell types between high-LRRN1 and low-LRRN1 groups in the GSE65194 dataset. **(H-K)** Detailed correlations between LRRN1 expression and immune cell infiltration. **(L-O)** Detailed correlations between LRRN1 expression and immune checkpoints. **(P-S)** Comparison of chemotherapeutic drug sensitivity between high and low LRRN1 expression groups in the GSE65194 dataset. “L”, Low expression of LRRN1; “H”, High expression of LRRN1.

To further evaluate the clinical therapeutic value of LRRN1 in breast cancer, we performed GSEA on the TCGA-BRCA cohort, stratified according to LRRN1 expression levels. This analysis revealed a significant association between high LRRN1 expression and the PD-L1/PD-1 checkpoint pathways (**Figures 6A, B**). External validation using the GEO database (GSE65194) corroborated these findings, demonstrating a positive correlation between LRRN1 and CD274 (**Figure 6C**) and a negative correlation with HHLA2 (**Figure 6D**). Moreover, estimation algorithms indicated significant correlations between LRRN1 expression and StromalScore, ImmuneScore and ESTIMATEScore (**Figure 6E**), implying altered dynamics within the TME. Heatmaps and bar charts further elucidated the relationships between LRRN1 expression and 22 immune cell types (**Figures 6F, G**), revealing a positive correlation with M1 macrophages and a negative correlation with M2 macrophages.

Additional scatter plots detailed the correlations between LRRN1 and various immune cells (**Figures 6H-K**), including T cells gamma delta (γδ T cell), resting dendritic cells (DCs), M1 macrophages, and M2 macrophages. These results reminded us to hypothesize that LRRN1 may influence the immune microenvironment of breast cancer by promoting M1 macrophage polarization. Furthermore, LRRN1 exhibited positive correlations with several immune checkpoint genes (**Figures 6L-O**), including CD48, CD86, TNFRSF14, and CD27 (p < 0.05), which may be functionally associated with M1 macrophages.

To explore the potential therapeutic value of LRRN1 in breast cancer, we analyzed drug sensitivity data from the GSDC database. The analysis revealed that high-LRRN1 subgroups exhibited significantly greater responsiveness to Lapatinib, Bortezomib, Axitinib, and Afatinib compared to low-LRRN1 subgroups, as evidenced by lower IC50 values (**Figures 6P-S**). Collectively, our findings suggest that LRRN1 activators may represent a promising therapeutic strategy for the treatment of breast cancer.

## Discussion

The metastatic cascade is a multifaceted and intricate process that encompasses a series of cellular events, such as primary tumor proliferation, invasion, immune evasion, and modulation of the tissue microenvironment (25). Additionally, the TME is crucial for cancer cell immune evasion and the progression of metastasis (26). In recent years, LRR protein family has emerged as a key focus in cancer metastasis research. The study reported LRRC45 facilitate lung cancer progression through the upregulation of c-MYC and several other proteins (27). Similarly, high expression of LRRC25 in gastric cancer has been reported to create an immunosuppressive microenvironment by modulating chemokine axes (28). In breast cancer, LRRC15 promotes the migration and invasion of triple-negative breast cancer (TNBC) cells via the Wnt/β-catenin signaling pathway (29). Furthermore, the LRRC15-ITGB1-FAK-PI3K signaling axis has been identified, which drives the progression of TNBC through a phosphorylation-dependent mechanism (30). Nevertheless, the mechanisms and functions of LRRN proteins in breast cancer remain largely unexplored, our study fills this gap by focusing on LRRN1, a previously understudied LRRN subfamily member, and revealing its tumor-suppressive role.

Previous studies have demonstrated that LRRN proteins exhibit context-dependent functions in cancer. LRRN4 has been shown to promote colorectal cancer malignancy through activation of the RAS/MAPK pathway (31), while LRRN3 has been associated with a lower risk of mesothelioma (32). Despite these insights, the clinical and functional significance of LRRN proteins in breast cancer have not been thoroughly investigated. Although LRRN1 overexpression has been previously associated with aggressive gastric cancer phenotypes and poor survival (9), our findings in breast cancer contrast sharply with this: we revealed an inverse correlation between LRRN1 expression and advanced pathological stages/TNM classifications, and survival analyses showed reduced LRRN1 expression significantly increased mortality risk (log-rank p < 0.01)—this tissue-specific difference in LRRN1 function highlights its context-dependent role, which has not been reported in prior LRRN1-related studies. Additionally, univariate and multivariate Cox regression analyses performed on the GEO dataset further corroborated these findings.

To elucidate the functional role of LRRN1, we employed a multi-faceted approach, including WGCNA, Pearson correlation analysis, and GSEA. By analyzing the intersection of enriched pathways, we pinpointed the Wnt signals as a potential downstream effector of LRRN1. We then conducted gain-of-function studies in breast cancer cells. Overexpression of LRRN1 markedly suppressed cellular migration, as demonstrated by functional assays and Western blot analysis. Mechanistic investigations through bioinformatics pathway enrichment implicated the Wnt signaling cascade as a potential downstream mediator of LRRN1’s effects. Wnt pathway activation, initiated by ligand-receptor interactions (e.g., Wnt3α/Wnt5α binding to Frizzled/LRP5/6 complexes), typically inhibits β-catenin degradation via the APC/GSK-3β/Axin complex (33, 34). Both canonical and non-canonical Wnt signaling pathways have been shown to contribute to EMT (35, 36). Notably, overexpression of LRRN1 increased the protein levels of Wnt5α/β, which aligns with prior studies demonstrating that Wnt5α inhibits breast cancer progression (37) and blocks cell migration via EMT-independent mechanisms (38)—our study extends this by identifying LRRN1 as a novel upstream regulator of Wnt5α, a link that has not been established in previous Wnt5α-related breast cancer research. This observation further supports a context-dependent tumor-suppressive role for LRRN1 in breast cancer.

Currently, immunotherapy has revolutionized the treatment landscape for many cancer types, including breast cancer. By enhancing the efficacy of the immune system in combating cancer, immunotherapy offers a durable therapeutic effect through immunologic memory (39). LRRN1 has been implicated in the PD-1/PD-L1 axis, a critical pathway in immune checkpoint regulation. Numerous studies have demonstrated that PD-1 inhibitors, such as pembrolizumab, atezolizumab, and tislelizumab, can be effectively combined with chemotherapy to treat metastatic TNBC (40, 41). HHLA2, also referred to as B7-H7, is primarily expressed in organs such as the breast, intestine, gallbladder, and placenta. Within the immune system, it is primarily detected in monocytes/macrophages and is widely present in various human tumor tissues, particularly in PD-1/PD-L1-negative tumors (42). Given its distinct immune evasion mechanisms from the PD-1/PD-L1 pathways, HHLA2 has emerged as a potential target for immunotherapy and revealed a worse prognosis (43). Our analysis indicated LRRN1 exhibits a positive correlation with CD274 (PD-L1) and a negative correlation with HHLA2 (*p* < 0.05). This suggests that LRRN1 may participate in the PD-1/PD-L1 immune checkpoint signals and could potentially augment the effectiveness of PD-1/PD-L1 blockade therapies in metastatic breast cancer. More importantly, additional scatter plots detailed the correlations between LRRN1 and various immune cells (**Figures 6F, G**), including γδ T cells, resting DCs, and M1/M2 macrophages (*p* < 0.001). Future research should explore the synergistic effects of LRRN1-targeted therapies in combination with existing immunotherapeutic strategies to improve outcomes for patients with metastatic breast cancer.

γδ T cells exhibit unconventional T-cell characteristics, such as MHC-independent antigen recognition and the ability to directly kill target cells via natural killer (NK) cell receptors (44). In breast cancer, recent studies have revealed that patients with high γδ T cell abundance are more likely to benefit from immunotherapy (45). Numerous studies have demonstrated that CD48 is expressed on various hematopoietic cells, including γδ T cells. γδ T cells can interact with CD48 through its receptor CD244, which is also expressed on these cells. CD48 crucial for the activation and regulation of both NK cells and γδ T cells via its interaction with CD244. These interactions are essential for immune surveillance, antiviral responses, and tumor immunity (46, 47). In our analysis, we observed a positive correlation with both γδ T cell abundance and CD48 expression in breast cancer. This suggests that LRRN1 may modulate the γδ T cells’ activity in the breast cancer immune TME, potentially influencing antitumor immunity. This extends prior findings on γδ T cells and CD48 by linking LRRN1 to this activation axis—unlike previous studies that only observed associations between γδ T cell abundance and immunotherapy response.

DCs captured tumor antigens from the surrounding microenvironment, process these antigens, and subsequently present them to naïve T cells in the lymph nodes. This process enables DCs to link the initial innate immune response with the subsequent adaptive immune response, thereby facilitating the differentiation of T cells into effector cells that can effectively target tumor cells (48). DCs constitute a functionally diverse group of professional antigen-presenting cells (APCs) that orchestrate adaptive immunity. The adoptive transfer of ex vivo activated and tumor antigen-loaded DCs, known as DC vaccination, has been demonstrated to induce cancer-controlling immune effector and memory responses in preclinical models (49). Additionally, promoting the maturation of DCs has been shown to promote the efficacy of cancer immunotherapy through PD-1/PD-L1 blockade, emerging as a promising strategy for immunotherapy (50, 51). DCs are among the primary cells expressing CD86. In their inactivated state, DCs exhibit relatively low surface expression of CD86, although CD86 mRNA is already present. Upon activation, the expression of CD86 on DCs is significantly upregulated (52). CD86 is a key regulator of T cell activation and immune responses, primarily through its interactions with CD28 and CTLA-4. These interactions render CD86 an important target for immunotherapeutic strategies (53). In our analysis, we found that a positive correlation between LRRN1 expression and both DCs abundance and CD86 expression. This suggests that LRRN1 may influence the activation and function of DCs within the TME, thereby modulating antitumor immune responses (55).

TME is a highly complex and dynamic ecosystem composed of various immune cells, fibroblasts, and a modified extracellular matrix that is rich in vasculature. Considering all these components, tumor-associated macrophages (TAMs) have been the focus of extensive research due to their critical roles in carcinogenesis, tumor progression, and immune evasion (54). TAMs exhibit a phenomenon termed “macrophage polarization,” differentiating into M1 or M2 macrophage phenotypes, each with distinct functions and implications for tumor biology (55). TAMs are composed of multiple heterogeneous subpopulations, including pro-inflammatory M1 macrophages and immunosuppressive M2 macrophages. M1 macrophages are characterized by their ability to promote inflammation and cytotoxicity, while M2 macrophages are associated with tissue repair, immune suppression, and tumor-supportive functions (55). The balance between M1 and M2 macrophages is critical, as an imbalance favoring M2 polarization is often linked to tumor progression (56). Importantly, TAMs also significantly influence the therapeutic efficacy of PD-1/PD-L1 inhibitors, highlighting their role in immune evasion and resistance to immunotherapy (55).

M1-type macrophages are characterized by their robust anti-tumor capabilities, which are achieved through the recognition and subsequent elimination of tumor cells. Two primary mechanisms have been identified through which M1 macrophages exert their cytotoxic effects on tumor cells: direct cytotoxicity and antibody-dependent cell-mediated cytotoxicity (ADCC) (57). In contrast to the anti-tumor activities of M1 macrophages, M2 macrophages are generally associated with pro-tumor functions. Specifically, M2 macrophages can degrade the basement membrane of endothelial cells by secreting a variety of proteolytic enzymes, including matrix metalloproteinases (MMPs), serine proteases, cathepsins, and other collagenases. These enzymes break down various collagens and other components of the extracellular matrix, thereby promoting the migration of tumor cells and tumor-associated stromal cells (55). In breast cancer, combination therapies have been shown to increase the abundance of M1 macrophages and activate CD8+ T cells and NK cells within the tumor, while significantly suppressing lung metastases. These findings suggest that the combination of CL7 and anti-PD-1 therapy has the potential to treat TNBC by remodeling the TME and inducing M1 macrophage polarization (58). Our analysis revealed that LRRN1 expression increased M1 macrophage polarization and reduced M2 macrophage polarization, indicated that LRRN1 could modulating the balance between M1 and M2 macrophages may be a potential therapeutic strategy for preventing metastasis, which was not reported in prior TAM-focused studies.

To further elucidate the clinical therapeutic potential of LRRN1 in breast cancer, a comprehensive analysis of relevant databases was performed. Our findings demonstrate significant associations between LRRN1 expression and the efficacy of several TKIs that are potentially used in breast cancer therapy such as Lapatinib, Bortezomib, Axitinib, and Afatinib (**Figures 6P-S**). These TKIs are often used in combination with chemotherapy, endocrine therapy, or other targeted agents due to challenges such as poor aqueous solubility, rapid clearance, or off-target toxicity (59–62). In our analysis, we observed that the IC50 values for these TKIs were markedly in the group with low LRRN1 expression compared to the group with high LRRN1 expression (*p* < 0.001). These results imply that tumors expressing high levels of LRRN1 may exhibit greater sensitivity to these TKIs. Notably, unlike prior studies, our findings position LRRN1 as a practical predictive marker—one that can stratify patients to avoid administering ineffective TKIs to those with low LRRN1 expression, thereby reducing unnecessary treatment-related toxicity. Furthermore, these analyses suggest that LRRN1 activator could potentially serve as synergistic agents to augment the effectiveness of TKI-based therapies. This potential extends not only to the specific TKIs examined in our study but also to other emerging TKIs that may be developed in the future. By modulating LRRN1 expression or activity, it may be possible to sensitize breast cancer cells to TKI treatment, thereby improving therapeutic outcomes.

## Conclusions

our analysis and experimental findings suggest that LRRN1 suppresses breast cancer metastasis and exhibits promising prognostic value for metastatic breast cancer. Furthermore, LRRN1 may participate in the activation of γδ T cells and T cells in TME. Additionally, LRRN1 could potentially modulate the M1/M2 balance macrophages in breast cancer TME, thereby influencing antitumor immune responses. Moreover, our findings indicate that LRRN1 activators could act as synergistic effectors to enhance the efficacy of TKI-based therapies. This potential extends not only to the specific TKIs examined in our study but also to other emerging TKIs that may be developed in the future. Further investigation into the mechanisms and functional confirmation of LRRN1 in breast cancer, particularly in TNBC, may provide valuable insights for developing novel immunotherapeutic strategies targeting this disease.

## Data Availability

The original contributions presented in the study are included in the article/**Supplementary Material**. Further inquiries can be directed to the corresponding author/s.

## Glossary

AJCC: American Joint Committee on Cancer
APCs: Antigen-Presenting Cells
DCs: Dendritic Cells
DFS: Disease-Free Survival
DMEM: Dulbecco’s Modified Eagle Medium
ECL: Enhanced Chemiluminescence
ER: Estrogen Receptor
FAK: Focal Adhesion Kinase
FBS: Fetal Bovine Serum
GDSC: Genomics of Drug Sensitivity in Cancer
GEO: Gene Expression Omnibus
GO: Gene Ontology
GSEA: Gene Set Enrichment Analysis
HER2: Human Epidermal Growth Factor Receptor 2
IC50: The half-maximal Inhibitory Concentration
KEGG: Kyoto Encyclopedia of Genes and Genomes
LRR: Leucine-rich Repeat
LRRC: Leucine-rich Repeat-containing
LRRN: Leucine-rich Repeat Neuronal
MMP2: Matrix Metalloproteinase-2
MMP9: Matrix Metalloproteinase-9
MRI: Magnetic Resonance Imaging
MTT: (3-(4,5-Dimethylthiazol-2-yl)-2,5-diphenyltetrazolium
NK: Natural Killer
NLRP3: Nod-like Receptor Protein 3
OS: Overall Survival
PBS: Phosphate Buffered Saline
PFA: Paraformaldehyde
PR: Progesterone Receptor
PS: Penicillin-Streptomycin
RFS: Recurrence-free Survival
RT-qPCR: RNA Extraction and Real-Time Quantitative Polymerase Chain Reaction
TAMs: Tumor-associated Macrophages
TBS: Tris-Buffered Saline
TCGA: The Cancer Genome Atlas
TME: Tumor Microenvironment
WGCNA: Weighted Gene Co-expression Network Analysis
γδ T cell: T cells gamma delta
